# The Impact of Specialist Palliative Care on the Utilization of Health Care Services at the End of Life Among Patients With Prostate Cancer: A Nationwide Register-based Study

**DOI:** 10.1016/j.euros.2026.03.003

**Published:** 2026-05-02

**Authors:** Timo P. Carpén, Nelli-Sofia A. Nåhls, Mikko S.J. Nuutinen, Tiina H. Saarto

**Affiliations:** a Palliative Care Center, Comprehensive Cancer Center, Helsinki University Hospital, and Faculty of Medicine, Helsinki University, Helsinki, Finland; b Department of Oncology, Vaasa, Central Hospital, The Wellbeing Services County of Ostrobothnia, Finland; c Department of Oncology, Comprehensive Cancer Centre and University of Helsinki, Helsinki, Finland; d Nordic Healthcare Group, Helsinki, Finland

**Keywords:** Emergency department, End-of-life care, Health care utilization, Prostate cancer, Specialist palliative care

## Abstract

**Background and objective:**

Prostate cancer is one of the leading causes of cancer mortality worldwide. Palliative care is essential for symptom management and quality of life. This study examined end-of-life (EOL) health care use among Finnish patients with prostate cancer and the association of timing of specialist palliative care (SPC) contact.

**Methods:**

Nationwide retrospective cohort study of all men who died of prostate cancer in 2019 (*n =* 921). Data from national registries. Patients categorized into Group I (SPC contact >30 d before death) or Group II (SPC contact ≤30 d/none).

**Outcomes:**

Emergency department (ED) contacts, hospitalizations, SPC use, and place of death. Multivariable logistic regression adjusted for age and municipality type.

**Key findings and limitations:**

Median age 81 yr. Only 30% of patients received SPC (median 101 d before death). In the last month, patients in Group I had fewer secondary hospitalizations (21% vs 38%, *p <* 0.001), lower readmissions (19% vs 26%, *p =* 0.035), and greater use of SPC inpatient unit (15% vs 2.1%, *p <* 0.001) and hospital-at-home (40% vs 4.4%, *p <* 0.001). Multivariable analysis: Group II had higher odds of ED contacts (odds ratio [OR] 1.46, 95% confidence interval [CI] 1.06–2.00) and secondary hospitalizations (OR 2.66, 95% CI 1.83–3.88). Limitations were retrospective design and no clinical data on disease severity or patient preferences.

**Conclusions and clinical implications:**

EOL health care use in prostate cancer is intensive. Patients with SPC contact more than 30 d before death had lower odds of acute hospital service use and higher odds of SPC service use during the last month of life compared with patients with later or no SPC contact. Findings support earlier SPC referral to improve patient-centered EOL care.

**Patient summary:**

Among Finnish men dying of prostate cancer, most used hospital services heavily and died in hospital. Earlier access to palliative care was associated with reduced acute health care use and increased care in SPC inpatient units.

## Take Home Message

Earlier specialist palliative care (SPC) contact in patients with prostate cancer was associated with reduced acute hospital use and increased access to SPC services. Timely referral improves patient-centered end-of-life care and should be integrated earlier into treatment pathways to enhance quality of care.

ADVANCING PRACTICE **What does this study add?**

Healthcare use at the end of life in patients with prostate cancer is intensive. This study showed that timely specialist palliative care contact had lower odds of acute hospital utilization and higher odds of palliative services. These nationwide findings support earlier referral to specialist palliative care to improve patient-centered end-of-life care.

**Clinical Relevance**

Earlier referral to specialist palliative care (SPC) is shown here to be associated with reduced emergency visits and hospitalizations in the final month of life of prostate cancer patients. Timely SPC involvement supports care in more appropriate settings, such as home or palliative units, aligning better with patient-centered goals. Avoiding burdensome hospital-based interventions near the end of life can reduce patient distress and improve quality of life. These findings highlight the importance of integrating SPC early to optimize care pathways and minimize unnecessary acute care use. Associate Editor: Roderick C.N. van den Bergh, MD PhD.

**Patient Summary**

Among Finnish men dying of prostate cancer, most used hospital services heavily and died in hospital. Earlier access to specialist palliative care (SPC) had lower odds of acute healthcare use and higher odds of care in SPC inpatient unit.

## 1 Introduction

Approximately 5500 new cases of prostate cancer are diagnosed annually in Finland and 1.5 million worldwide [1], [2]. Prostate cancer has remained one of the world’s leading causes of cancer death among men, with 397 000 deaths in 2022 [2], and in Finland with 900 deaths annually [1]. In addition to the high mortality, patients with advanced prostate cancer suffer from a high symptom burden. Compared to men with localized disease, men with advanced prostate cancer experience greater pain and fatigue, higher levels of psychosocial distress, increased risk of suicide, and poorer health-related quality of life (QOL) [3], [4], [5].

Specialist palliative care (SPC) is multidisciplinary and comprehensive care aimed at improving symptom control and QOL [6]. In several randomized controlled trials, early involvement of SPC has been shown to be effective in improving symptom burden and QOL for patients with advanced solid tumors [7], [8], [9], [10]. Therefore, international cancer organizations, including the American Society of Clinical Oncology and the European Society for Medical Oncology, recommend the involvement of palliative care early in the disease trajectories of patients with advanced cancer [11], [12].

In addition to the benefits in QOL, palliative care has been demonstrated to reduce health care utilization, such as hospital admissions and emergency department (ED) visits at the end of life (EOL) among patients with advanced cancer [13], [14], [15], [16], [17]. Banner et al [18] showed that use of acute health care resources is common among patients with advanced prostate cancer during the final year of life as patients had four hospitalizations on average, with a median duration of 39 d, and approximately two-thirds of the patients died in hospital. Despite the high health care utilization among patients with advanced prostate cancer, there have been no nationwide reports to date on the association of SPC services on health care utilization at the EOL among this population.

This study aimed to investigate the utilization of health and social care services at the EOL among patients who died of prostate cancer in Finland, and to examine whether the timing of the first SPC contact was associated with acute health care use during the last 30 d of life.

## 2 Materials and methods

### 2.1 Cohort selection

The cohort comprised all adult (≥18 yr old) patients who died of prostate cancer in Finland between January 1, 2019, and December 31, 2019. The patients were identified nationwide from the 2019 Causes of Death Register (Statistics Finland) according to the International Classification of Disease, Tenth Revision (ICD-10) coding for prostate cancer (C61) as the primary cause of death. The study was conducted in accordance with the Strengthening the Reporting of Observational Studies in Epidemiology (STROBE) guidelines [19].

### 2.2 Data collection

The National Care Register and Kanta Services are comprehensive registries established by Finnish health care authorities. These registries are regulated by law, requiring participation from all health care providers, including both public and private sectors. The data on health care and social service utilization were extracted from these databases.

The data collection is explained in detail in our previous publication [20]. The data collection was conducted from the beginning of 2018 until the end of 2019.

### 2.3 Utilization of health and social care services

In Finland, primary health care is mainly organized by municipalities and delivered by general practitioners. Secondary health care is provided by 20 district hospitals and tertiary care by five university hospitals across the entire country. In this study, given the purposes of the analyses, secondary and tertiary health care are combined and referred to as “secondary health care”. This study included data on hospital admissions, ED contacts, outpatient clinic contacts, social services, and home care.

All health care utilization outcomes were evaluated during the last 30 d of life and were defined as binary variables (≥1 event vs none), in line with established EOL quality indicators. ED contact was defined as at least one ED contact. Outpatient clinic contacts were defined as at least one outpatient contact in either primary or secondary care. Hospitalizations were defined as at least one inpatient admission, separately for primary care and secondary care. Readmissions were defined as at least one readmission during the last 30 d of life and were evaluated separately for ED readmission, all-cause hospital readmissions, secondary care hospital readmissions, and primary care hospital readmissions.

### 2.4 Palliative care

In Finland, palliative care services are divided into general and specialist level, of which the latter, that is, SPC, is offered in primary care and secondary care, whereas general PC is offered only in primary care. In this study, we focused on SPC services, defined as a service conducted by palliative care specialists in the following settings: outpatient clinics, hospital-at-home services, SPC inpatient units or hospices, and inpatient consultations. Each encounter, whether an outpatient visit, hospital-at home contact, inpatient consultation, or admission to a specialized inpatient unit or hospice, counted as one SPC contact.

Based on the timing of the first SPC contact, patients were classified into two groups as follows: (1) Group I (the first contact with SPC occurred more than 30 d before death) and (2) Group II (the first SPC contact occurred either in the last 30 d before death or not at all). Patients who received SPC ≤30 d before death were grouped together with those who did not receive SPC, as SPC initiated during the last 30-d outcome window cannot influence health care utilization across the entire last 30 d of life. The 30-d cutoff was chosen in advance because SPC initiated within the last 30 d is unlikely to affect health care utilization throughout the entire last month of the outcome window.

From the data, ICD-10 code Z51.5 was collected, which refers to a palliative care decision—a decision when curative or disease-modifying treatments are discontinued—and the focus shifts primarily toward palliative care with intent to improve symptom control and QOL.

### 2.5 Place of death

Place of death was obtained from the 2019 Cause of Death Register data with the following classifications: home, long-term care facility, or hospital (including primary health care and secondary hospitals). Place of death was reported as SPC inpatient unit if the patient was hospitalized in an SPC inpatient unit on the day of death.

### 2.6 Ethical statement

The study was conducted with the Finnish Institute for Health and Welfare (THL) and approved by THL Dnr: THL/908/6.02.00/2021. According to the Finnish legislation for research, there was no need for a separate ethics committee approval, as the data used in the study comprised deceased patients.

### 2.7 Statistical analyses

For the statistical analyses, we used IBM-SPSS version 29 (IBM Corp, Armonk, NY, USA). Descriptive statistics are presented as medians and interquartile ranges (25th–75th percentiles) for continuous variables and as numbers and percentages for categorical variables. Study groups were defined according to the timing of first SPC contact.

The primary outcomes were ED contacts and secondary care hospitalizations during the last 30 d of life. Secondary outcomes included utilization of SPC services and place of death. Descriptive analyses were used to characterize health care utilization during the last 30 d of life according to the timing of SPC contact.

For categorical analyses, Pearson’s chi-squared test and Fisher exact tests were used. Given the unequal distributions, Mann-Whitney test was used to analyze differences in hospital days between different groups. Logistic regression analyses were performed to identify independent factors associated with ED contacts and hospitalizations during the last 30 d of life. Variables included in the multivariable models were age at death (modeled as a continuous variable and scaled per 10-yr increase), municipality type (categorized as urban, semi-urban, rural), and timing of SPC contact. A *p* value of <0.05 was considered statistically significant.

## 3 Results

### 3.1 Patient characteristics

The study included 921 patients with a median age of 81 yr (interquartile range 74–87) at the time of death. In total, only 30% (*n =* 276) used SPC services. The median interval from the first SPC contact until death was 101 d. According to the timing of SPC contact, patients were classified into Group I (*n =* 215, 23%) and Group II (*n =* 706, 77%). Patients in Group I were younger (median 78 vs 81 yr, *p <* 0.001), more likely to live in urban areas (78% vs 55%, *p <* 0.001), and more often had a documented palliative care decision (76% vs 41%, *p <* 0.001). The patient characteristics are presented in the Table 1.

**Table 1:** Patient characteristics

Number of patients (%)	Group I (*n =* 215)	Group II (*n =* 706)
Age in yr median (IQR)	78 (72–85)	81 (74–87)
Municipality type		
Urban	168 (78)	390 (55)
Semi-urban	20 (9.3)	152 (22)
Rural	27 (13)	164 (23)
ICD-10 Diagnosis code Z51.5 Palliative care	163 (76)	291 (41)
SPC contact	215 (100)	61 (8.6)
Median time from the first SPC contact to the death (IQR)	131 d (81–273)	14 d (7–23)

IQR = interquartile range; *n =* number of patients; SPC = specialist palliative care. Group I = first SPC contact >30 d before death; Group II = first SPC contact ≤30 d before death or no contact.

### 3.2 Health care and social service utilization

In the last month of life, patients in Group I were associated with lower health care utilization compared to patients in Group II. Patients in Group I had fewer secondary care hospitalizations (21% vs 38%, *p <* 0.001) and secondary care outpatient visits (32% vs 50%, *p <* 0.001), and higher use of SPC inpatient units (15% vs 2.1%, *p <* 0.001) and hospital-at-home services (40% vs 4.4%, *p <* 0.001). There was no statistically significant difference in ED contacts between Group I and Group II (45% vs 53%, *p =* 0.052). The results of the health care utilization are presented in the Table 2.

**Table 2:** Health care and social service utilization during the last month of life according to the timing of the first specialist palliative care (SPC) contact

Number of patients (%)	Group I (*n =* 215)	Group II (*n =* 706)	*p* value
Emergency department contacts	97 (45)	372 (53)	0.052
Outpatient clinic contacts			
Secondary health care	69 (32)	352 (50)	<0.001
Primary health care	150 (70)	498 (71)	0.828
Hospitalizations			
Hospitalizations all	141 (66)	526 (75)	0.010
Secondary health care	44 (21)	265 (38)	<0.001
Primary health care	120 (56)	417 (59)	0.397
Readmissions			
Emergency department readmissions	46 (21)	165 (23)	0.546
All-cause hospital readmissions	40 (19)	181 (26)	0.035
Secondary care hospital readmission	14 (6.5)	87 (12)	0.017
Primary care hospital readmission	29 (14)	116 (16)	0.300
Specialist palliative care			
Palliative care outpatient unit	38 (18)	26 (3.7)	<0.001
Hospital-at-home	85 (40)	31 (4.4)	<0.001
Specialist palliative care inpatient unit	33 (15)	15 (2.1)	<0.001
Social services	32 (15)	152 (22)	0.033
Home care	111 (52)	325 (46)	0.150
Place of death			
Home	33 (15)	74 (11)	0.051
Hospital-at-home SPC	20 (9.3)	8 (1.1)	<0.001
Hospital	141 (66)	530 (75)	0.006
Long-term care	13 (6.0)	88 (13)	0.008
Specialist palliative care inpatient unit	28 (13)	14 (2.0)	<0.001

*n =* number of patients. Group I = first SPC contact >30 d before death; Group II = first SPC contact ≤30 d before death or no contact. All outcomes are reported as the proportion of patients with ≥1 event during the last 30 d of life. *p* values were calculated using Pearson’s chi-square test or Fisher’s exact test for categorical variables, and the Mann-Whitney U test for continuous variables. SPC = specialist palliative care.

Most patients (73%) died in hospital. A minority died at home (12%), in long-term care facilities (11%), or in SPC inpatient units (4.6%). Patients in Group I were more likely to die in an SPC unit (22%), either in an SPC inpatient unit or at home with the support of an SPC hospital-at-home team, compared with 3.1% in Group II (Table 2).

### 3.3 Factors associated with emergency department contacts and hospitalizations

Multivariable logistic regression adjusting for age and municipality type showed that patients in Group II had higher odds of ED visits (odds ratio [OR] 1.46; 95% confidence interval [CI] 1.06–2.00; *p =* 0.019) and secondary care hospitalizations (OR 2.66; 95% CI 1.83–3.88; *p <* 0.001) in the last month compared to patients in Group I.

Age was not significantly associated with ED contacts or secondary care hospitalizations. Semi-urban residence was associated with lower odds of secondary care hospitalizations compared with urban residence (OR 0.67; 95% CI 0.46–0.99) (Table 3).

**Table 3:** Factors associated with emergency department contacts and hospitalizations in secondary care during the last 30 d of life: logistic regression analysis

A. Emergency department contacts			
	OR	95% CI	*p* value
Age (per 10-yr increase)	0.91	0.71–1.17	0.461
Municipality type			0.173
Urban	Reference		
Semi-urban	0.74	0.53–1.06	
Rural	0.80	0.58–1.12	
Timing of specialist palliative care contact			0.019
Group I	Reference		
Group II	1.46	1.06–2.00	
**B. Secondary care hospitalizations**			
Age (per 10-yr increase)	0.92	0.71–1.20	0.545
Municipality type			0.123
Urban	Reference		
Semi-urban	0.67	0.46–0.99	
Rural	0.88	0.62–1.26	
Timing of specialist palliative care contact			<0.001
Group I	Reference		
Group II	2.66	1.83–3.88	

*n* = number of patients; SPC = specialist palliative care; OR = odds ratio; CI = confidence interval. Group I = first SPC contact >30 d before death; Group II = first SPC contact ≤30 d before death or no contact.

### 3.4 Hospital readmissions

Patients in Group II were associated with more frequent hospital readmissions in the last month of life (26% vs 19%, *p =* 0.035), particularly in secondary care hospitals (12% vs 6.5%, *p =* 0.017) (Table 2). No significant differences were found in ED readmissions or primary care readmissions.

## 4 Discussion

This nationwide cohort study revealed high utilization of health care services among patients with advanced prostate cancer during their final months of life, with most patients frequently using ED and secondary health care services. SPC was used by only a minority of patients, even though SPC contact in Group I was significantly associated with reduced health care utilization, particularly fewer secondary care hospitalizations, and outpatient contacts, and greater use of SPC inpatient unit and hospital-at-home services at the EOL. These findings emphasize the potential benefits of timely SPC initiation in optimizing EOL care, and reducing acute hospital use for patients with advanced prostate cancer.

These observations are consistent with prior studies showing that men with advanced prostate cancer frequently experience intensive EOL care and hospital deaths. In our nationwide cohort, 86% had at least one ED contact and 65% were hospitalized in secondary care during the last 6 mo. In comparison, a recent register-based analysis by Banner et al. [18] reported that men with advanced prostate cancer had on average four hospitalizations during their final year of life, with a median duration of 39 inpatient days. Regarding place of death, 73% of men in our cohort died in hospital, which is similar to the findings by Banner et al, where nearly two-thirds of patients died in hospital [18].

Taken together, these results mirror each other and underscore how resource-intensive EOL trajectories remain in this disease. Similar trends have also been observed in other cancer populations, with substantial health care utilization and aggressive treatments common near the EOL [21], [22], [23]. Although the timing of SPC was dichotomized, the 30-d cutoff is clinically meaningful and consistent with established EOL quality indicators as the last month prior death is the most resource-intensive period [14], [20], [24].

Despite the symptom burden and guideline recommendations, SPC remains underused in this population. In our cohort, only 30% of patients received SPC services, with a median of 101 d before death, which can be considered a moderate level of timely access compared with many reports of very late initiation [25], [26]. However, this is clearly lower than in the recent Swedish registry study by Bergqvist et al, where 76% of patients with prostate cancer received SPC during the last 3 mo of life [25]. Similarly, population-based data including all cancer patients have shown that only about one-quarter received SPC, and half of this initiated contact within the last 30 d of life, highlighting substantial delays in routine practice [26]. Our data extend these observations by demonstrating geographic variation within Finland, with greater access in urban municipalities as SPC services were mainly concentrated in larger cities at the time of the study period.

We explicitly classified timing of SPC using a 30-d threshold because late initiation is closely linked to markers of overly aggressive EOL care. Earle et al proposed and validated claims-based quality indicators including chemotherapy within 14 d of death, intensive care use, ED visits, and hospitalizations in the last 30 d as signals of suboptimal EOL care [22], [24], [27]. Building on this framework, studies have used ≤30 d before death to denote “late” palliative involvement [14]. Using the same cutoff point in our analysis facilitates comparability and focuses attention on the window most strongly associated with avoidable high-intensity acute care.

Timely SPC contact in our cohort was associated with lower use of secondary care in the last month, fewer hospitalizations and outpatient visits, fewer ED contacts, and greater utilization of SPC inpatient units and hospital-at-home services. Observational studies have similarly associated timely palliative input with lower ED visits, fewer readmissions, and fewer hospital deaths [14], [25], [28]. In our study, patients in Group II had 1.5-fold higher odds of ED contacts and more than 2.7-fold higher odds of secondary care hospitalizations compared with those with patients in Group I. Group II is heterogeneous, including both patients who received SPC very late and those who did not receive SPC at all, and these subgroups may differ in frailty and health care trajectories. These findings may reflect differences in care pathways, advance care planning, symptom burden, frailty, and access to SPC services between the groups.

The high rate of hospital deaths (73%) in our cohort contrasts with the preferences many patients express for dying outside hospital, and also with health-system aims to support home or hospice deaths when desired [29]. Timely SPC contact was associated with more deaths in SPC inpatient units and fewer hospital deaths, aligning with registry evidence from Sweden that specialized palliative involvement is associated with fewer ED visits and hospital deaths [25].

### 4.1 Limitations

This study benefits from comprehensive nationwide coverage, capturing all deaths due to prostate cancer in Finland in 2019, which strengthens its generalizability within the Finnish health care context. However, as a retrospective registry-based study, it cannot establish causality. Although we adjusted for age and municipality type, residual confounding remains possible, particularly due to the lack of data on clinical factors such as disease severity, functional status, cultural background, comorbidities, overall disease burden, and survival time from diagnosis. In addition, information on socioeconomic factors was not available. These unmeasured factors may have influenced both access to SPC and health care utilization, which may limit the generalizability of the findings to health care systems with similar organizational structures.

In addition, the definition of Group I requires patients to survive long enough after SPC initiation, introducing potential survival bias and the possibility of reverse causality. Data on concurrent disease-modifying or life-prolonging prostate cancer treatments were not available, which may have influenced health care utilization patterns and has, therefore, been acknowledged as a limitation. Continuous variables were modeled using linear terms. Potential nonlinearity of age was assessed using a spline model and did not improve model fit.

Finally, multiple comparisons were performed, particularly in descriptive analyses, which increases the risk of type I error (alpha inflation). Therefore, findings from unadjusted comparisons should be interpreted cautiously and primarily considered hypothesis-generating rather than confirmatory. In addition, to verify the clinical significance of our results, prospective studies are warranted in the future.

## 5 Conclusions

EOL health care utilization among patients with prostate cancer was intensive. This study showed that timely SPC contact had lower odds of acute hospital service use and higher use of SPC services during the last month of life. These findings support earlier integration of SPC into prostate cancer care pathways to improve patient-centered EOL care.

  ***Author contributions:*** Timo P. Carpén, Nelli-Sofia A. Nåhls, Mikko S.J. Nuutinen had full access to all the data in the study and takes responsibility for the integrity of the data and the accuracy of the data analysis.

  *Study concept and design:* Carpén, Nåhls, Saarto.

*Acquisition of data:* Carpén, Nåhls, Nuutinen.

*Analysis and interpretation of data:* Carpén, Nåhls, Nuutinen

*Drafting of the manuscript:* Carpén, Nåhls.

*Critical revision of the manuscript for important intellectual content:* Carpén, Nåhls, Nuutinen, Saarto

*Statistical analysis:* Carpén, Nåhls, Nuutinen

*Obtaining funding:* Carpén, Nåhls, Nuutinen, Saarto

*Administrative, technical, or material support:* Carpén, Nuutinen, Saarto

*Supervision:* Saarto

*Other* (specify): None

  ***Financial disclosures:*** Timo P. Carpén certifies that all conflicts of interest, including specific financial interests and relationships and affiliations relevant to the subject matter or materials discussed in the manuscript (eg, employment/affiliation, grants or funding, consultancies, honoraria, stock ownership or options, expert testimony, royalties, or patents filed, received, or pending), are the following: None.

  ***Funding/Support and role of the sponsor*:** The study was funded by the Cancer Foundation Finland and Helsinki University Hospital, Comprehensive Cancer Center, State Research Funding. Open access funded by Helsinki University Library.

  ***Data availability:*** As the data is part of the larger dataset owned by the Finnish Institute for Health and Welfare (THL), the data generated during the current study is not publicly available. However, the data is available from the principal author Tiina Saarto upon reasonable request and with permission of the THL.

  ***Ethics approval and consent to participate:*** Informed consent: Not applicable, as the study included only deceased patients.

  ***Ethics approval:*** This was a retrospective nationwide register-based study using data from the Finnish National Care Registers and the Kanta Services concerning patients who died of prostate cancer in 2019. The study was conducted in collaboration with the Finnish Institute for Health and Welfare (THL) within the Project on Quality Information on Palliative and End-of-Life Care and approved by THL (Dnr: 12345556). In accordance with Finnish research legislation, additional approval from an ethics committee was not required because the study population consisted solely of deceased individuals. All data were pseudoanonymized prior to analysis. All methods were performed in compliance with the relevant regulations and guidelines.
